# Physicochemical Characteristics of Model Membranes Composed of *Legionella gormanii* Lipids

**DOI:** 10.3390/membranes13030356

**Published:** 2023-03-20

**Authors:** Katarzyna Pastuszak, Elżbieta Chmiel, Bożena Kowalczyk, Jacek Tarasiuk, Małgorzata Jurak, Marta Palusińska-Szysz

**Affiliations:** 1 Department of Interfacial Phenomena, Institute of Chemical Sciences, Faculty of Chemistry, Maria Curie-Skłodowska University, Maria Curie-Skłodowska Sq. 3, 20-031 Lublin, Poland; kasia_pastuszak98@wp.pl; 2 Department of Genetics and Microbiology, Institute of Biological Sciences, Faculty of Biology and Biotechnology, Maria Curie-Skłodowska University, Akademicka 19, 20-033 Lublin, Poland; e.chmiel@sp27.lublin.eu (E.C.); b.kowalczyk746@wp.pl (B.K.); jacek_tarasiuk@onet.pl (J.T.); marta.palusinska-szysz@mail.umcs.pl (M.P.-S.)

**Keywords:** *Legionella gormanii*, phospholipids, Langmuir monolayer technique

## Abstract

*Legionella gormanii* is one of the species belonging to the genus *Legionella*, which causes atypical community-acquired pneumonia. The most important virulence factors that enable the bacteria to colonize the host organism are associated with the cell surface. Lipids building the cell envelope are crucial not only for the membrane integrity of *L. gormanii* but also by virtue of being a dynamic site of interactions between the pathogen and the metabolites supplied by its host. The utilization of exogenous choline by the *Legionella* species results in changes in the lipids’ composition, which influences the physicochemical properties of the cell surface. The aim of this study was to characterize the interfacial properties of the phospholipids extracted from *L. gormanii* cultured with (PL+choline) and without exogenous choline (PL−choline). The Langmuir monolayer technique coupled with the surface potential (SPOT) sensor and the Brewster angle microscope (BAM) made it possible to prepare the lipid monomolecular films (model membranes) and study their properties at the liquid/air interface at 20 °C and 37 °C. The results indicate the effect of the choline addition to the bacterial medium on the properties of the *L. gormanii* phospholipid membranes. The differences were revealed in the organization of monolayers, their molecular packing and ordering, degree of condensation and changes in the components’ miscibility. These findings are the basis for further research on the mechanisms of adaptation of this pathogen, which by changing the native composition and properties of lipids, bypasses the action of antimicrobial compounds and avoids the host immune attack.

## 1. Introduction

The cell envelope is a complex multilayered structure that, in most bacteria, is essential for the maintenance of cell shape and structural integrity. The Gram-negative bacterium cell envelope consists of the outer (OM) and inner (IM) membranes. The periplasm with the peptidoglycan cell wall and unique proteins separate these membranes. The OM is an unusual lipid bilayer that contains phospholipids in the inner leaflet and glycolipids, principally lipopolysaccharides (LPS), in the outer leaflet [1]. This asymmetric nature and the unique biochemistry of LPS contribute to the ability of OM to function as a molecular permeability barrier that protects the bacterium, confining the access of noxious compounds present in the environment. The macromolecules of the cell envelope play a central role in the properties and capabilities of the cell. This holds particularly true for the pathogenic bacteria whose highly specific interactions with the human organism depend on surface structures to a large extent. *Legionella* are Gram-negative bacteria that are ubiquitous in freshwater reservoirs, soils, and water-based engineered structures. Human infection leads to legionellosis, which can be present in its non-pneumonic (Pontiac fever) and acute pneumonic (Legionnaires disease) forms. The ability of *Legionella* to overcome the killing mechanisms of phagocytes depends on many factors, both the specialized systems of protein secretion and the components related to the unique structure of their cell envelope.

*L. gormanii* is one of the species belonging to the genus *Legionella*, which causes atypical community-acquired pneumonia in immunocompromised and immunocompetent individuals [2,3]. Rare cases of *L. gormanii*-induced pneumonia were diagnosed in children with immunodeficiency [4]. The pathogenicity of this bacterium is determined, among others, by the high efficiency of pulmonary surfactant degradation comparable to the activity of *L. pneumophila*. *L. gormanii* bacteria produce phospholipase B, for which the substrate is phosphatidylcholine (PC), accounting for 80% of the lung surfactant [5].

This bacterium does not reproduce in *Acanthamoeba* spp., but it has the ability to adhere, invade and replicate in human macrophages of the THP-1 line [6]. The proteins secreted by the T4 secretion system play a key role in the formation of a distinct membrane-bound replicative niche by *L. pneumophila* inside the macrophages. The bioinformatic analyses of the *L. gormanii* genome revealed the presence of a gene cluster encoding the T4SS components [7]. The activity of membrane proteins, their folding, stability or localization are controlled by the protein–lipid interactions regulated by phospholipids [8].

The *L. gormanii* membranes were characterized by the unique bacterial lipid composition. The lipid profile of the OM and the IM *L. gormanii* comprised glycerolipids (triglycerides, diglycerides), sphingolipids (ceramides, hexosylceramides) and the most abundant, phospholipids (phosphatidylethanolamine, PE; phosphatidylcholine, PC; cardiolipin, CL; phosphatidylglycerol, PG). The presence of PC and ceramides—the lipids commonly occurring in the eukaryotic cells and rarely found in the prokaryotic organisms—is a unique feature of *L. gormanii* membranes. This bacterium can synthesize PC via two independent pathways, namely the phospholipid N-methylation (Pmt) pathway and the phosphatidylcholine synthase (Pcs) pathway. The bacteria condense choline with CDP-DAG by the Pcs synthase in the Pcs pathway. Since choline is not a biosynthetic product of bacteria, its source for *L. gormanii* are metabolites derived from the eukaryotic cells. In this way, these bacteria come into close contact with the host cell in which they replicate. A rapid PC synthesis (in a one-step reaction) may be required for the *L. gormanii* bacteria to adjust their membrane physiology rapidly to new environmental conditions. *L. gormanii* cultured on the medium with exogenous choline was synthesized by 21% more PC and less PE and CL (12% and 9%, respectively) compared with the bacteria grown on the medium without the addition of choline [9]. Our previous lipidomic analyses showed quantitative differences in the molecular profile of the various phospholipids contained in the OM and IM of *L. gormanii* grown on the choline-supplemented and non-supplemented media. The content of PE15:0_15:0, PE15:0_16:0 and PE16:0_16:1 was higher in the IM lipids extracted from the bacteria cultured with the choline addition. The amount of PC cyclopropyl17:0_15:0 and PC cyclopropyl17:0_16:0 in the IM lipids isolated from the bacteria grown on the medium with exogenous choline was higher compared to the IM lipids isolated from *L. gormanii* cultured without choline. The OM lipids from the bacteria grown on the choline-supplemented medium contained more PC15:0_15:0 than the bacteria grown on the medium without exogenous choline [10]. The results indicate that the lipid membranes are a dynamic site of interactions between the bacterial pathogen and the choline delivered into the growth medium. 

The structure of lipids, mainly the length and degree of saturation of the phospholipids’ hydrocarbon tails, influence the physicochemical properties of the membrane that determine bacterial survival and the adaptation to the living environment. One of the simple and at the same time the most precise methods for monitoring the physicochemical properties of membranes is the Langmuir technique. This technique enables the formation of high-quality, ordered monolayers that can be used as a model for bacterial membranes. The use of the Langmuir technique in combination with the Brewster angle microscope allows for the precise imaging of the monolayer morphology upon the film compression. This study aimed at determining how the changes in the structure of phospholipids isolated from the bacteria cultured with and without the exogenous choline affect the biophysical properties of *L. gormanii* membranes using the Langmuir monolayers. In addition, the use of the Brewster angle microscope allowed for a more detailed analysis of the film architecture and visualization of the interfacial organization of PL constituents of the monolayer. These studies will provide the basis for further analysis of the effectiveness of interactions of various antimicrobial compounds with the biological systems obtained from *L. gormanii* grown under different culture conditions.

## 2. Materials and Methods

### 2.1. Bacterial Strain and Media

*Legionella gormanii* (ATCC 33297) was cultured on the buffered charcoal yeast extract (BCYE) agar plates (Oxoid, Basingstoke, UK) or on this medium enriched with 100 µg/mL choline chloride (Sigma-Aldrich, St. Louis, MO, USA; referred to as choline in this paper) at 37 °C and 5% CO_2_ in a humid atmosphere for 3 days. Biomass was harvested by centrifugation at 8000 rpm, 20 min, washed with 0.5 M NaCl twice, distilled water once and then freeze-dried.

### 2.2. Extraction and Separation of Phospholipid by TLC Plates

Lipids were extracted from 500 mg of freeze-dried bacterial cells according to the procedure of Bligh and Dyer [11]. The extracts were concentrated in a vacuum evaporator until complete dryness and resolved in 100 μL of chloroform/methanol (1:2; *v*/*v*). The organic layer contained lipids and pigments, mainly legioliulin, which is responsible for the blue-white autofluorescence under the long-wavelength UV light [12]. The phospholipids (PLs) were purified from the pigment using one-dimensional thin-layer chromatography (TLC) on the silica gel 60 F254 plates of the size 10 cm × 10 cm (Merck, Darmstadt, Germany). Before the application of the organic phase, the plates were washed with chloroform/methanol (1:1, *v*/*v*) to remove all contaminants and dried at room temperature. The organic fraction (about 2 mg) was applied on the silica gel as a narrow band and developed with the solvent system chloroform/methanol/acetic acid (98:2:1, *v*/*v*/*v*). PLs were visualized with iodine vapor, and legioliulin was detected under the long-wavelength UV (Transiluminator UV-953). The PL-containing band was scraped off, transferred to the screw-capped tubes and extracted from the silica gel with a mixture of chloroform/methanol (1:1, *v*/*v*). PLs purified from the pigment were intended for testing in the Langmuir trough and separation into individual classes.

Phospholipids were separated into different classes using chloroform/methanol/acetic acid (13:5:2, *v*/*v*/*v*) as the solvent system. Four bands were detected after the iodide vapor exposure; they were scraped and extracted with chloroform/methanol (1:1, *v*/*v*). The identification of individual PLs was made comparing the retention factor (Rf) coefficients of individual components with the Rf values for standard phospholipids (Rf,_PC_ = 0.09, Rf,_PE_ = 0.37, Rf,_PG_ = 0.56, Rf,_CL_ = 0.79). The Rf value of a PL was equal to the distance traveled by the compound divided by the distance traveled by the solvent front. Separations of PLs were made in six replications. Lipids from all fractions were collected, evaporated to dryness under nitrogen, weighed and stored at −20 °C before the analysis of fatty acids. To assess the reproducibility of the results, three independent culturing experiments for each condition with and without the choline supplementation were analyzed.

### 2.3. Preparation of Fatty Acid Methyl Esters (FAMEs)

A phospholipid sample (1 mg) in the Teflon-capped Pyrex tube was hydrolyzed with 1 mL of 0.8 M NaOH in 50% methanol at 80 °C for 1 h. After cooling to room temperature, the reaction mixture was acidified with 200 µL of 6 M HCl, evaporating to dryness under a nitrogen stream at 40 °C. The released free fatty acids (FFAs) were extracted with 1 mL of chloroform. The chloroform solution of FFAs was dried with anhydrous sodium sulfate and the chloroform was removed under the nitrogen stream. The FFAs were methylated with 300 µL of 0.02 M trimethylsilyl diazomethane (TMSD, Sigma-Aldrich) in 20% methanol in acetone at room temperature for 30 min. The excess solvent and diazomethane were removed under a gentle stream of nitrogen. A total of 2 mL of water and 1 mL of chloroform were added to the sample. After thorough mixing, the sample was centrifuged at 5000× *g*, for 10 min, and the FAMEs dissolved in the chloroform layer were analyzed by gas–liquid chromatography and mass spectrometry (GLC/MS).

### 2.4. Gas-Liquid Chromatography and Mass Spectrometry

FAMEs were analyzed using a gas chromatograph (Agilent Technologies, Santa Clara, CA, USA, instrument 7890A) connected to a mass selective detector (Agilent Technologies MSD 5975C, inert XL EI/CI) (GLC-MS), using helium as a carrier gas. The chromatograph was equipped with an HP-5MS column (30 m × 0.25 mm). The temperature program was as follows: 150 °C for 5 min raised to 310 °C (5 °C/min), and the final temperature was maintained for 10 min. FAMEs were identified by an analysis of their mass spectra and fragmentation patterns. The relative content (%) of each fatty acid was calculated from the ratio of the area of its peak to the total area of all peaks. The positions of the branching methyl group, cyclopropane ring and the double bonds were determined by an analysis of the mass spectra of fatty acid pyrrolidines.

On the basis of the relative content of identified fatty acids, the molecular weight of the particular phospholipid classes, and then the total value for the mixture was calculated according to the following equation (Equation (1)):

(1) $$\bar{M}=M_{1}x_{1}+M_{2}x_{2}+\ldots +M_{n}x_{n}$$

where $\bar{M}$ is a mean molecular weight, M is a molecular weight of a given compound and x is a molar fraction of a given compound in the mixture.

The obtained results are presented in Table 1. They were used in the Langmuir monolayer studies.

### 2.5. Langmuir Monolayer Studies

The Langmuir (KSV NIMA) and Langmuir–Blodgett (KSV 2000 Standard) troughs equipped with symmetrical barriers and a Wilhelmy plate were used for the surface pressure-mean molecular area (π−A) isotherm determination. The accuracy of the pressure sensor was 0.1 mN/m. For all the experiments, the trough was filled with 0.01% acetic acid solution prepared by diluting the concentrate one (99.7%) with Milli-Q water purified by the Milli-Q Plus system (resistivity 18.2 MΩ cm). The temperature was kept constant (20 or 37 °C) by the external water circulating system (Lauda). Phospholipids (PLs) isolated from *L. gormanii* cultured with or without choline were prepared in chloroform/methanol (4:1, *v*/*v*) to obtain the concentration of 1 mg/mL. The solvents of high purity (≥99%) were purchased from Avantor Performance Materials Poland S.A. (Gliwice, Poland). The determined molecular weights (Table 1) were used to estimate the number of molecules to be deposited on the subphase. To achieve this, the correct volume of a phospholipid solution with known concentration (usually 1 mg/mL) was dropped carefully onto the subphase (0.01% acetic acid) with the glass-made Hamilton microsyringe and left for 10 min to ensure full evaporation of the volatile solvents. Then, the monolayer was continuously compressed at the rate of 10 mm/min to its collapse. Each experiment was performed at least three times and the obtained π−A isotherms were found to be reproducible with the average error of ±2 ${Å}^{2}$/molecule. Simultaneously, the surface potential-mean molecular area (ΔV−A) isotherms were recorded by means of vibrating plate surface potential sensor (SPOT, Biolin Scientific, Gothenburg, Sweden) with the accuracy of 1 mV.

The monolayer morphology was directly visualized with the help of Brewster angle microscope (nanofilm_ultrabam, Accurion, Göttingen, Germany). The use of 50 mW internal solid-state laser emitting p-polarized light with the wavelength of 658 nm enabled the recording of BAM images with the lateral resolution of 2 μm and the field of view of 720 × 400 μm^2^. The angle of incident was fixed to the Brewster angle (53.2°).

### 2.6. Statistical Analysis

The values were expressed as the mean ± SD for three independent experiments. The results were statistically evaluated using the one-sided asymptotic Mann–Whitney U test with the continuity correction (implemented in Python’s 1.10.0 version of the Scipy package). The significance level was set to 5%.

## 3. Results

### 3.1. Fatty Acid Composition of Phospholipids

**Figure 1:**
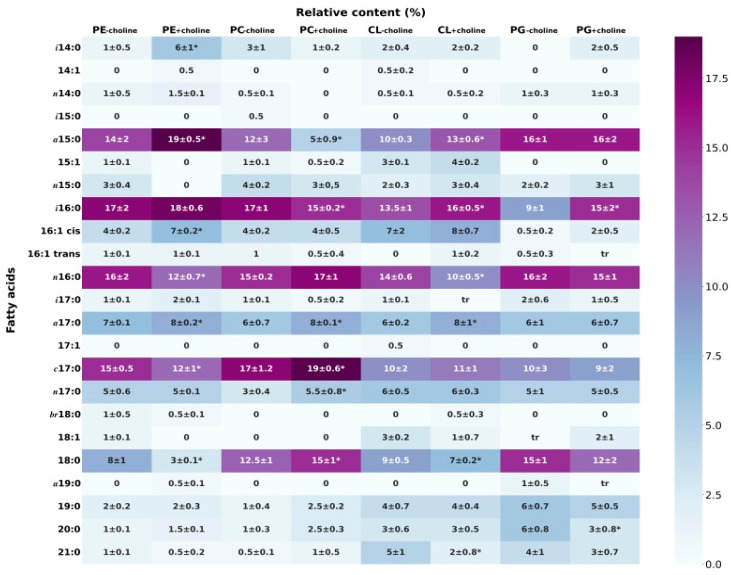
Relative content (%) of fatty acids in the PL classes isolated from the bacteria cultured on the medium without and with choline. *a*, methyl branch at the anteiso carbon atom; *i*, methyl branch at the iso carbon atom; *n*, unbranched acid; *c*, cyclopropane ring structure; ± standard deviation; * indicates a significant difference in the MW test for choline vs. the lack of choline comparison in each PL. The test was carried out for fatty acids with at least 5% relative content.

The composition of fatty acids, which are the main components of lipids, determines the physical, chemical and physiological properties of each class of phospholipids. Our previous research proved that the *L. gormanii* phospholipid classes have characteristic FA distributions [10]. In the present studies, it is shown that adding choline to the growth medium changes the content of unsaturated, saturated and long-chain fatty acids in *L. gormanii* phospholipids (Figure 1 and Table 2). The bacteria grown on the medium with choline on the average synthesized 2.4 times more long-chain (from C19 to C21) fatty acids, less saturated fatty acids and fatty acids with the acyl chain length from C14 to C18 found in the PC class. On the contrary, *L. gormanii* grown on the medium with choline synthesized less saturated and long-chain fatty acids, and more unsaturated fatty acids in the PG class. A similar pattern of fatty acids was found in the PE class of the bacteria grown on the choline-supplemented and non-supplemented medium. In the CL class, the content of saturated and unsaturated fatty acids was similar regardless of the culture conditions. However, the addition of choline to the growth medium caused these bacteria to synthesize 3% less long-chain fatty acids and 3% more fatty acids with the acyl chain length of C14 to C18. Additionally, there were minor differences in the share of saturated fatty acids between the culture conditions, but none was significant.

### 3.2. π−A Isotherms and Compression Modulus

**Figure 2:**
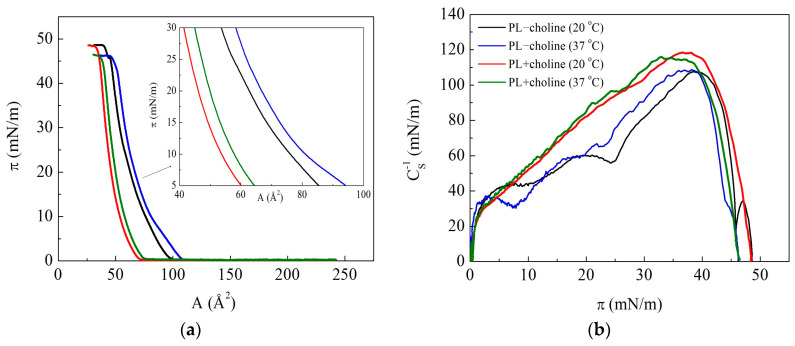
Surface pressure-area per molecule (π−A) isotherms registered at 20 °C and 37 °C (**a**) and determined the compression modulus-surface pressure ($C_{S}^{-1}-\pi$) dependencies based on them (**b**) for the monolayers of phospholipids (PL) extracted from *L. gormanii* bacteria supplemented (PL+choline) or not (PL−choline) with choline.

Phospholipids extracted from the *L. gormanii* bacteria, both supplemented and non-supplemented with choline, form Langmuir monolayers at the air–liquid interface at 20 °C and 37 °C. The obtained π−A isotherms are presented in Figure 2a. 

Based on them (Figure 2a), the lift-off point ($A_{0}$) and the collapse pressure ($\pi_{c}$) were determined. The lift-off point ($A_{0}$) is the area per molecule at which the surface pressure can be detected (≈0.5 mN/m). It is ascribed to the monolayer transition from the gas to liquid-expanded phase. The collapse pressure ($\pi_{c}$), at which a sudden change in the slope of the π−A isotherm is observed, corresponds to breakdown of the two-dimensional monolayer structure and formation of three-dimensional aggregates (collapsed domains) and/or loss of molecules from the monolayer by their dissolution in the subphase. The values of the above-mentioned parameters ($A_{0}$ ± 2$\;{Å}^{2}$/molecule, $\pi_{c}$ ± 0.1 mN/m) are summarized in Table 3 for measurements conducted at 20 °C and 37 °C.

A difference between the PL−choline and the PL+choline monolayers concerns the course of π−A isotherms, both their shape and area per molecule at a given surface pressure and temperature. As regards to the shape, the π−A isotherm of the PL+choline monolayer displays a moderate increase in the surface pressure with compression, without any visible discontinuities, while that of the PL−choline monolayer displays subtle inflections as changes in the curve slope at the surface pressure of about 8 mN/m (at 37 °C) or 10 mN/m (at 20 °C) and 25 mN/m (at both). These can be indicative of a change in the arrangement and packing of molecules in the PL−choline monolayer which does not occur for PL+choline. At 37 °C, the π−A isotherms are slightly shifted toward larger molecular areas (to the right side of the x axis) and the film collapse pressure is lower than at 20 °C. Moreover, the lift-off point and the area per molecule in the full range of the surface pressures are lower for PL+choline than for PL−choline, suggesting a more condensed character of the former monolayers (Table 3). This fact correlates with a larger content of cardiolipin in PL−choline (21%) and can be a result of the anionic character of this phospholipid as well as its large headgroup, which is discussed in the following part of this paper.

To confirm the above assumption, the compression modulus ($C_{S}^{-1}$, Figure 2b) was calculated directly from the π−A isotherm data based on the following formula [13]:

(2) $$C_{S}^{-1}=-A{\left(\frac{d\pi }{dA}\right)}_{T,p}$$

The compression modulus is a typical parameter providing information about packing and ordering of the monolayer at constant temperature (T) and external pressure (p). The determined values of $C_{S}^{-1}$ as a function of π are presented in Figure 2. Moreover, the maximal $C_{S}^{-1}$ values along with the surface pressure and mean molecular area at which they occur are listed in Table 3.

The maximal values of $C_{S}^{-1}$ for both PL−choline and PL+choline monolayers are within the range assigned to the liquid-condensed (LC) state according to the Davies and Rideal criterion, i.e., 100 mN/m < $C_{S}^{-1}$ < 250 mN/m [13]. However, up to the collapse pressure, the $C_{S}^{-1}$ values for PL+choline are greater than for PL−choline at both temperatures, thus revealing a tighter packing of molecules. It should be emphasized that the course of the $C_{S}^{-1}=f\left(\pi \right)$ function determined for PL−choline shows the presence of two distinct minima (inflections) at about 8–10 mN/m and 25 mN/m, which corresponds to the discontinuities on the π−A isotherms (Figure 2). The $C_{S}^{-1}$ values of the peaks rise from the range characteristic of the LE (40 mN/m) through the LE-LC (60 mN/m) to the LC (108 mN/m) phase for the monolayer at 20 °C. As a contiguous LE monolayer is created, the π−A isotherm takes off, giving rise to a positive surface pressure. Then, the hydrophobic tails of the molecules come into contact with each other by being largely disordered and fluid [14]. Since there is the change in the physical state of the monolayers from the LE to the LC phase, the first inflection therefore proves that the LE–LC phase transition takes place. However, it is not typical of a first-order phase transition represented by a horizontal *plateau* in the π−A isotherm and nearly zero compression modulus [15]. It is blurred due to the multicomponent composition of the PL−choline monolayer whose molecules contain different headgroups and chains. The second inflection indicates further alterations in the film organization, but it is difficult to unequivocally determine their origin. A few options can be considered: (1) a second-order phase transition related to the changes in alkyl chain tilting; (2) partial miscibility and/or removal of some molecules into the subphase as molecules can be forced out of the interface; (3) some nucleation process [15].

In the case of PL+choline monolayers, the π−A isotherms demonstrate no visible inflections, and even after differentiation, no discontinuities are observed on the compression modulus. This indicates that, during the compression, the PL+choline film is more tightly packed than PL−choline, while remaining in the same physical state.

These findings show that the choline addition to the bacteria medium increases the condensation of the PL+choline monolayers as manifested by larger values of the compressibility modulus than those of PL−choline. The reason for that can be stronger Lifshitz-van der Waals forces between the molecules possessing longer acyl chains. Moreover, the hydrophilic headgroup sizes are of great importance, which is discussed below.

### 3.3. Surface Potential

**Figure 3:**
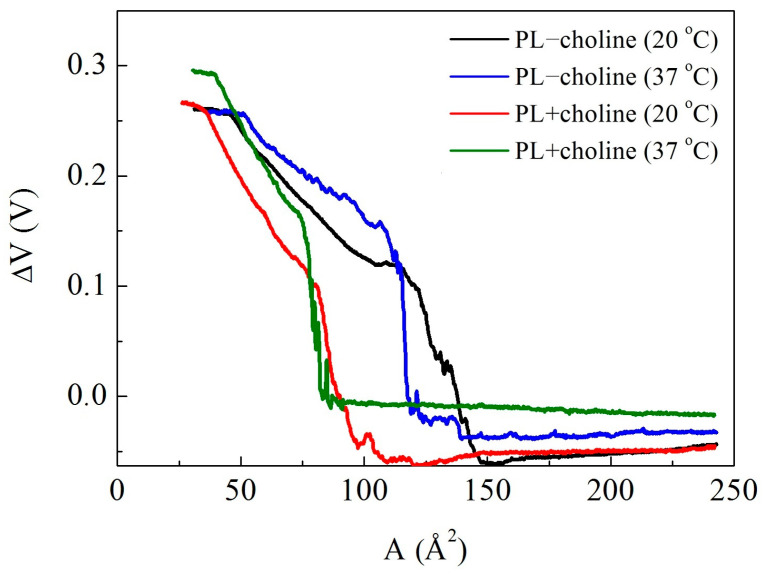
Surface potential changes ΔV vs. the mean molecular area (A) for the PL−choline and PL+choline monolayers at 20 °C and 37 °C.

The surface potential changes (ΔV) reveal the alteration of orientation and conformation of molecules in the monolayers upon compression. Measured ΔV defines the difference between the surface potential of pure subphase and the surface with the monolayer [16]. The alterations of the normal component of the dipole moment density with respect to the surface, caused by phospholipid film compression, result in proportional surface potential change, which allows one to analyze molecular behavior [17]. As can be seen in Figure 3, the ΔV values of all monolayers are positive upon compression. Variations in the slope of ΔV−A isotherms reflect molecular orientation and/or conformational changes in the monolayer since ΔV is proportional to the magnitude of the electrostatic field gradient perpendicular to the subphase surface [17,18]. During the compression, the hydrocarbon chains orient more vertically to the surface, changing the orientation of the polar groups, which contributes to an increase in the surface potential. Figure 3 shows a systematic increase in the ΔV values with the decreasing area per molecule of PL films in the range from 0 to 0.3 V.

At 37 °C, the ΔV changes are adequate, but the values of this parameter are greater than at 20 °C. It is also important to note that the sharp increase in the surface potential corresponds to the extensive surface coverage with the condensed domains, as revealed by the BAM images (see Section 3.4). When the PL−choline films are compressed up to the close-packed state, their ΔV values reach ~0.26–0.27 V, while for PL+choline it is 0.27–0.30 V.

**Figure 4:**
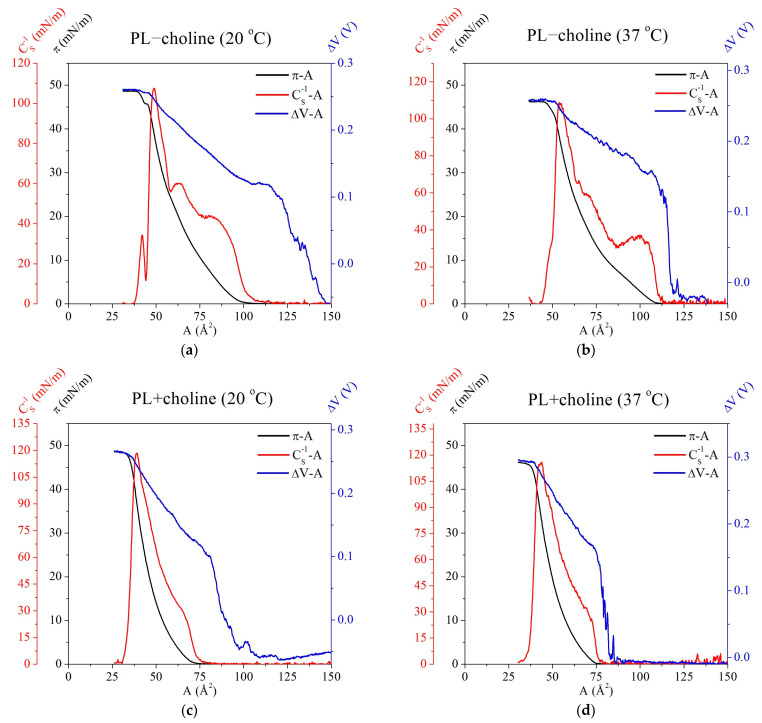
Surface pressure (π), compressibility modulus ($C_{S}^{-1}$) and surface potential changes (ΔV), vs. the mean molecular area (A) for the obtained monolayers: PL−choline at 20 °C (**a**), PL−choline at 37 °C (**b**), PL+choline at 20 °C (**c**) and PL+choline at 37 °C (**d**).

For better visualization, the correlation between the determined parameters, i.e., surface pressure (π), compressibillity modulus ($C_{S}^{-1})$ and surface potential changes (ΔV) as a function of area per molecule (A), is presented in Figure 4. A sharp increase in the ΔV values is found at larger areas than the lift-off point for the π−A isotherm. At certain area values, the ΔV increases more slowly. It is important to note that this inflection correlates with the π−A isotherm lift-off area. This suggests a change in the molecule orientation with respect to the subphase surface, related to the formation of more ordered structures. Moreover, the maximal values of the surface pressure usually correspond to the closest packing of molecules in the monolayer [18,19], which in the presented research can be observed as a strict correlation between the maximal values of ΔV and $C_{S}^{-1}$ as they occur at nearly the same area per molecule in all analyzed monolayers (Figure 4).

### 3.4. Morphology

**Figure 5:**
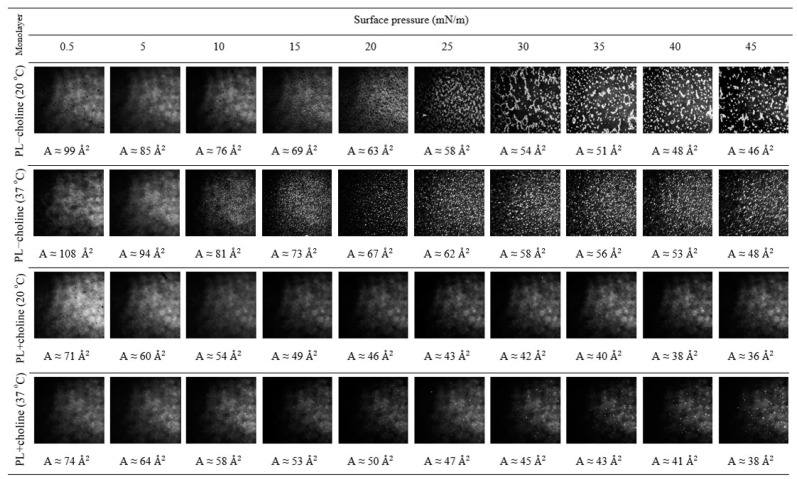
BAM images taken for the PL−choline and PL+choline monolayers at 20 °C and 37 °C at given values of surface pressure and mean molecular area.

The above observations can be evidenced by the BAM images presented in Figure 5. They depict that, during the compression of the PL−choline monolayer at 20 °C, some inhomogeneity appears at the surface pressure around 10 mN/m, after the reorganization indicated by the π−A isotherm. Namely, the coexistence of bright areas of more condensed domains (irregular oblong structures) surrounded by darker regions of the less condensed phase (patches) is observed. At about 25 mN/m the condensed domains are being restructured to larger ones, which is demonstrated by a small reduction in the compression modulus (Figure 2b, second inflection). At 37 °C, the domains are smaller in size and with the compression form regular circular structures densely distributed over the subphase. They appear in greater quantities at smaller values of surface pressure as compared to the analysis conducted at a lower temperature, and their reorganization at 25 mN/m is less pronounced at a temperature higher than 20 °C, which is also reflected in a smaller decrease in the compression modulus values (Figure 2b). Simultaneously, the changes in the monolayer organization correlate with the above-mentioned inflections of the π−A isotherms (Figure 2a).

Considering the morphology of the PL+choline monolayers, one can see much smaller and fewer circular domains, causing the film to be more homogeneous. At a lower temperature, more condensed structures can be clearly observed at about 25 mN/m, while at 37 °C a few domains can be noticed around 10 mN/m. However, their presence is not manifested either in the π−A isotherm inflections or in the discrete course of the $C_{S}^{-1}-\pi$ functions. Thus, the BAM images confirm the greater homogeneity of the PL+choline monolayers. This correlates with the greater condensation of the monolayers indicated by the higher $C_{S}^{-1}$ values.

## 4. Discussion

In this contribution, we studied the properties of model membranes (Langmuir monolayers) composed of lipids isolated from the *L. gormanii* cells grown on the medium with and without choline. The presence of choline affects the fatty acid composition (Figure 1) and proportions of the individual classes of phospholipids produced by bacteria (Table 2) and thus, the properties of the monolayers formed from them at the liquid–gas interface as well. The relative content of individual lipid classes shows that the zwitterionic phospholipids PE and PC dominate, the total amount of which is 76% for *L. gormanii* (−choline) and 85% for *L. gormanii* (+choline), while the negatively charged lipids (PG and CL) account for 24% and 15%, respectively [9]. These differences are of great importance for the model membrane behavior and intermolecular interactions in the phospholipid mixture, as can be seen on the π−A isotherms (Figure 2a).

**Figure 6:**
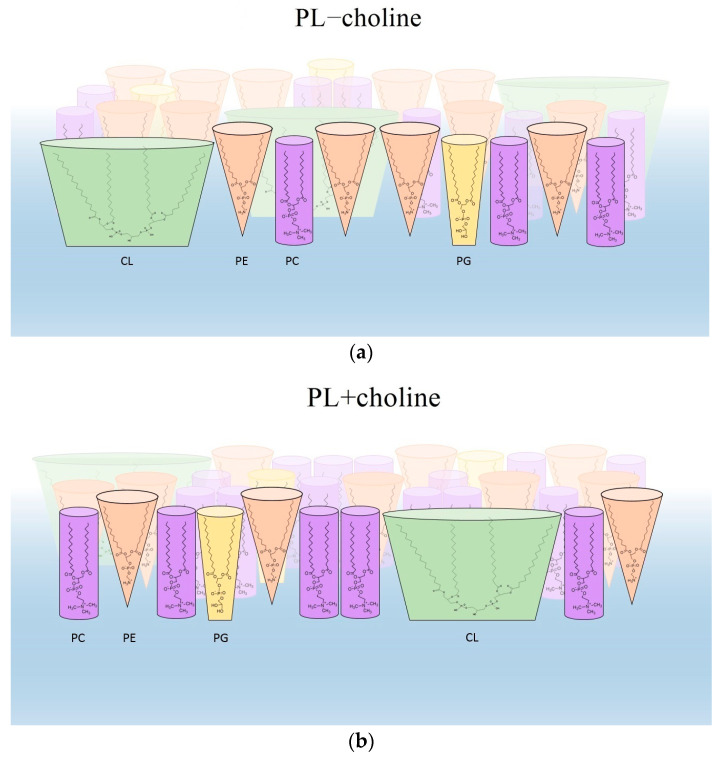
The effect of molecule shape on packing of the mixed monolayers, composed of phospholipids isolated from *L. gormanii* bacteria cultured without (**a**) and with (**b**) choline.

Comparing the π−A isotherms registered for the monolayers of PL−choline and PL+choline mixtures at 20 °C, it can be found that, in principle, the curves obtained for PL+choline are located at smaller areas per molecule than the π−A isotherms acquired for PL−choline (Figure 2a). This suggests that the distance between the PL+choline molecules is much smaller than between the PL−choline molecules. This fact correlates well with the higher values of the compressibility modulus for the PL+choline than for PL−choline monolayers, clearly indicating that the PL+choline monolayer is more packed at both temperatures (Figure 2b). A tighter packing of molecules contributes to the increased ordering of the acyl chains. This can be associated with the synergistic effect of Lifshitz-van der Waals (mainly dispersion) interactions that are larger for longer alkyl chains, C19–C21 (Table 2), fostering an easier ordering of molecules in the PL+choline monolayer under the compression. Such behavior is confirmed by the morphology images showing more homogeneous phase (in comparison to PL−choline, Figure 5), due to the increased packing density and miscibility of the compounds. Instead, in the PL−choline monolayers, the decisive factor for packing seems to be the presence of shorter C14–C18 unsaturated chains, which provokes a fluidizing disorder in the hydrophobic region, as well as higher CL percentage (21%). Furthermore, headgroup type and size are of great importance, as they determine the shape of the molecule. Small headgroup or large hydrophobic region result in a more conical shape [20], causing steric effects and affecting intermolecular interactions (Figure 6).

In PL+choline high percentage of PC, cylindrical-shaped phospholipid, results in a greater condensation of the molecules in the monolayer [21]. On the contrary, as mentioned, the PL−choline mixture contains more CL and PE (Table 2). Due to head-tail mismatch in these compounds, greater steric effects occur, and therefore a lower degree of packing is observed (Figure 6). The increased expansion in the headgroup region of this monolayer is revealed by the smaller values of $C_{S}^{-1}$ with respect to PL+choline (Figure 2b). Another cause of the decreased elasticity can be the reorganization of the monolayer detected by the domains’ formation.

Since PE and PC quantitatively constitute the largest fraction of the studied PL, it is important to analyze intermolecular interactions between them and changes in films caused by the altered amounts of these compounds in the mixed phospholipid monolayers. In the mixtures, the electroneutral PC and PE molecules separate the PG and CL molecules, thus reducing the repulsion between them [22,23,24]. The PC group is of particular importance in this study, as choline is added to the medium for the bacteria culture. The polar phosphocholine (PC) group consists of negatively ($-{\mathrm{OPO}}_{3}^{-}-$) and positively ($-N^{+}{\left({\mathrm{CH}}_{3}\right)}_{3}$) charged moieties separated by two $-{\mathrm{CH}}_{2}-$ groups. Such a separation allows for the formation of two ionic forms. The first one involves the maximal distance of charges, whereas the other one involves the reduced distance due to the internal salt linkage between the ionic charges in the PC head [25]. In the pH range of 2–8, the PC-containing monolayers do not bear an electric charge, which means that the phosphate and trimethylammonium groups neutralize each other, and this neutralization depends on the intermolecular spacing in the monolayers [25]. The molecular simulation study of the PC/PE mixtures [26] showed that most of the PC headgroups point toward the aqueous phase, while the majority of the PE headgroups point toward the hydrophobic core. Thus, the PE headgroups are probably localized closer to the fatty acid chain region, resulting in the closer packing of PL molecules. Therefore, the PC groups more exposed to the aqueous phase are more flexible.

When the PL+choline molecules become closer to each other during the compression, the intermolecular spacing decreases. The simulations conducted by Leekumjorn and Sum showed that the PC group becomes more aligned with the normal membrane due to the close packing of the PL molecules (smaller area/headgroup) [26]. This contributes to the ionic repulsion between similar charges of the polar groups and/or H-bonds formation, weakening the internal salt linkage. In contrast to PC, the PE groups can form inter- and intramolecular hydrogen bonds where the amine group interacts favorably with the phosphate/carbonyl moieties or water. Presumably, the PL monolayer compression provokes a change in the orientation of the headgroup from horizontal to more vertical. This is particularly observed for the PL+choline monolayer where, in consequence, a smaller area per molecule than in the PL−choline monolayer was obtained (Figure 2a). The heads become more aligned with the normal to membrane due to the close packing of the PL molecules. In contrast, it is likely that the heads in PL−choline monolayers are more horizontally oriented, so the distance between the phosphate groups is greater. If the area per headgroup is larger than the minimum cross-sectional area of two hydrocarbon chains measured perpendicularly to the tail direction, the chains must be tilted to some extent to compensate for the head-tail mismatch and thus maximize the contact in order to form a stable monolayer at the liquid-air interface [27,28]. Probably, such an inclination takes place in PL−choline molecules which thereby occupy larger areas, and the corresponding π−A isotherm is shifted to the right (Figure 2a). Furthermore, the ionic repulsions between the polar groups are reduced, whereas the intermolecular hydrogen bonds are weaker (or their total number decreases) and/or the internal salt linkage is strengthened.

A higher content of negatively charged molecules in the PL−choline film (24%) likely contributes to the limited miscibility of the components and greater expansion of the monolayer than that of PL+choline, where the PG and CL contents are much smaller (15%). This effect can be attributed specifically to the increased CL content in the PL−choline mixture as the PG content is the same in both cases. As already mentioned, cardiolipin has a specific, dimeric structure and the molecule is rather large in comparison to the other analyzed phospholipids, which causes the sterical effects [29]. It has been noted that it also causes less condensation of the films [23], so it is justified to assume that in addition to the FA composition (the presence of short chains), it is partially responsible for smaller compressibility modulus values for PL−choline than for PL+choline (Figure 2b). Moreover, this weakens the interactions between the other components of the monolayer, such as PE and PG [29]. Beyond the CL percentage, the reorganization of the monolayer revealed in the formation of condensed domains and/or the dissolution of some molecules into the subphase may contribute to the increased expansion and the differences between the analyzed films.

It is worth mentioning that the domains occur naturally in the bacterial membranes to minimize the system energy [30]. A key role in their formation is ascribed to the phospholipid composition and stronger interactions between particular components, which allow for sequestering preferentially into distinct regions of the cell [31]. As a result, the domains enriched in some of the components are segregated from the rest of the membrane depleted in these phospholipids which, beyond composition, also differ in the degree of order. The appearance of such inhomogeneity is manifested in the BAM images (Figure 5). Thus, the presence of domains demonstrates the partial miscibility of the compounds in the monolayers and the formation of regions with greater or lesser condensation (phase coexistence) depending on the monolayer composition. In the case of monolayers of phospholipids with the same headgroups in the molecules, the presence of domains results from the variation in the length and proportion of the saturated and unsaturated FA chains, which also has an effect on the mixed monolayer structure. Contrary to the phospholipids with unsaturated fatty acids, the phospholipids with saturated FAs tend to be close to each other, forming more condensed microdomains of relatively high molecular ordering [32], which are visible as brighter regions in the BAM images. This corresponds well to the condensed areas present in the PL−choline films (Figure 5), in contrast to PL+choline. As mentioned above, the saturated and long chains form favorably more condensed domains due to stronger Lifshitz-van der Waals interactions between fatty acid chains [18]. Moreover, the important factor in the domain formation seems to be the presence of phospholipids with negative curvature tendency, such as PE and CL [30]. Due to the shape of the molecules (Figure 6), the compounds can be separated to the concave-shaped areas of the model membrane [30], which differentiates the surface morphology in these areas. As PL−choline contains 21% of CL and 50% of PE, it is justified to assume that large, condensed regions are a consequence of curvature properties of the mentioned phospholipid classes. Smaller contents of CL (9% less) and PE (12% less) in PL+choline, in comparison to PL−choline, contribute to the better miscibility (Figure 5) of the phospholipid molecules as well as a higher level of condensation (Figure 2b and Figure 6). As the temperature increases, the breaking of Lifshitz-van der Waals forces between the hydrocarbon chains converts them into a much more fluid and disordered state with the increased cross-sectional area per lipid [33]. Moreover, the dipole-dipole interactions within the headgroup region can result in the weakening of in-plane polar attractions and contribute to lower homogeneity occurring at 37 °C (Figure 5).

Molecular reorganization and domain formation can be accompanied by the compensation of the dipole moments (monolayer depolarization) [17,34] as evidenced by the slope of ΔV−A isotherm changes (Figure 3). It is worth highlighting that the polarization of carbonyl groups >C=O within the PL heads makes a positive contribution to the ΔV values. The other groups are of less importance because they are submerged in the aqueous solution, so they are highly shielded due to the high dielectric constant and the conductivity of the subphase. Thus, the >C=O groups located in the hydrophobic chain region are the potential determining groups. Fluctuations in the surface potential at large areas or nearly zero surface pressure are likely due to the formation of small domains within the mostly gas phase monolayer (Figure 3). At very low surface pressures (π ≈ 0 mN/m), the *plateau* in π−A isotherm corresponds to the G-LE phase transition, where LE domains can be seen in the BAM images (not shown). By coalescing the domains to the expanded films (as the transition from gas (G) to the liquid-expanded (LE) state occurs), ΔV increases (Figure 3) abruptly, which is associated with a change in the slope of molecules from the horizontal to more vertical position with respect to the subphase plane [35]. The monolayer begins to form a more organized structure due to a larger amount of molecules in the close proximity being able to interact with one another, which can be observed as the sharp ΔV increase. This potential rise corresponds to the so-called critical area, which indicates the point when the hydrogen bonds with the water molecules are broken and the monolayer becomes structured [19,36,37]. This entails changes in the orientation of the intrinsic molecular dipole of the molecules in the film and of the water molecules near the monolayer interface [17]. With the structuring of the monolayer at the critical area, water is probably removed from the headgroup/subphase interface since the H-bonded network is broken. Therefore, the dielectric constant of such an interface is decreased sharply. This causes the increase in the surface potential [36,37]. However, a change in the surface pressure is not observed at the critical area as the accuracy of the surface pressure measurements is not enough to detect such a change [36]. Moreover, the critical area is larger for the PL−choline monolayer as presumably its molecules are involved in the H-bonds with water to a greater extent than those of PL+choline. The reason for that can be the increased amount of PE in PL−choline (Table 2), which exhibits a particular ability to interact strongly through inter- and intramolecular hydrogen bonds with both the other phospholipids and water [27]. A similar interpretation of the critical area increase by involvement in the hydrogen bonding with water molecules was applied by Brekiesz et al. for perfluorodecyldecane and its derivatives [18].

As mentioned above, as molecules (FA chains and headgroups) change their orientation to a more vertical one, they force the variation in the > C=O groups’ positioning, causing the surface potential increase (Figure 3). It is also important to mention that the surface potential values are higher at 37 °C compared to 20 °C. The reason for this phenomenon is the greater thermal motion of the molecules. As the temperature rises, phospholipid molecules have a larger kinetic energy, resulting in a lower ordering of molecules [38].

In summary, the PL+choline monolayer is found to be more densely packed and ordered owing to favorable interactions via the H-bonding and Lifshitz-van der Waals forces between the headgroups and long fatty acid chains C19–C21, respectively, as hydrocarbon chains are more perpendicular to the liquid-air interface. The intermolecular forces between the molecules in the PL−choline monolayers can be less attractive, due to large CL and short-chain FA contents, leading to lower miscibility and domain formation.

Analyzing the changes in monolayer behavior caused by external factors allows for a better understanding of the physiology of *L. gormanii* bacteria and therefore possibly leads to the development of new infection treatments in the future. The ordering and packing of monolayers, which are a model of bacterial membranes, can determine the way they interact with drugs and antimicrobial peptides, such as the LL-37 peptide [39,40].

## 5. Conclusions

The aim of this study was to determine how the ability of using extracellular choline for the PC synthesis in the Pcs pathway affects the physicochemical characteristics of model membranes composed of *L. gormanii* phospholipids by means of the Langmuir monolayer technique coupled with the surface potential sensor and Brewster angle microscope. The following conclusions can be drawn:

1. External conditions, such as temperature or presence of choline, have an impact on the bacterial membrane composition and in consequence, the physicochemical properties of the cell.
2. The obtained results exhibited differences in the content of fatty acids present in individual phospholipids extracted from bacteria cultured on a medium with and without choline. These differences made it possible to explain the changes in the degree of packing and ordering of monolayers.
3. The physical state of phospholipid monolayers can determine the interactions with external factors acting on bacteria. The denser packing and ordering of *L. gormanii* membranes composed of PLs extracted from bacteria grown on the medium with choline can suggest that such bacteria will be more resistant to the bactericidal action of agents inside the nutrient-rich but hostile environment of the host.
4. Domain formation of different condensation and composition can be of great importance for *L. gormanii* membrane functioning, including proper activity of proteins, and developing the interaction mechanisms with the host cell and antibacterial substances.

## Figures and Tables

**Table 1:** Molecular weight of phospholipid classes and their mixtures.

**Phospholipid Class**	**Molecular Weight (g/mol)**
PC−choline	730.6
PC+choline	729.4
PE−choline	683.5
PE+choline	680.9
CL−choline	1389.7
CL+choline	1385.9
PG−choline	715.8
PG+choline	714.0
**Phospholipid Mixture**	**Molecular Weight (g/mol)**
PL−choline	845.0
PL+choline	789.3

**Table 2:** Fatty acid (FA) composition (relative content, %) of individual phospholipids (this study). The percentage of individual classes in the phospholipid mixture isolated from *L. gormanii* cultured on the medium without and with choline (previous study [9]); * indicates a significant difference in the MW test for choline vs. the lack of choline comparison in each PL.

	Sum of Saturated FA	Sum of Unsaturated FA	Sum of FA 14–18	Sum of FA 19–21	Percentage of Phospholipid Class in PL Mixture [9]
PC−choline	77.0 ± 0.6	23.0 ± 0.5	97.5 ± 0.7	2.5 ± 0.3	26.0 ± 2.0
PC+choline	75.0 ± 0.5	24.0 ± 0.4	94.0 ± 0.5 *	6.0 ± 0.3 *	47.0 ± 0.0
PE−choline	79.0 ± 0.7	21.0 ± 0.2	96.0 ± 0.7	4.0 ± 0.1	50.0 ± 1.4
PE+choline	79.5 ± 0.3	20.5 ± 0.3	95.5 ± 0.4	4.5 ± 0.2	38.0 ± 0.6
CL−choline	76.0 ± 0.5	24.0 ± 0.8	88.0 ± 0.6	12.0 ± 0.8	21.0 ± 1.4
CL+choline	75.0 ± 0.4	25.0 ± 0.6	91.0 ± 0.5 *	9.0 ± 0.6 *	12.0 ± 0.6
PG−choline	89.0 ± 0.9	11.0 ± 1.0	83.0 ± 1.0	17.0 ± 0.6	3.0 ± 0.7
PG+choline	87.0 ± 1.0	13.0 ± 1.0	89.0 ± 1.0 *	11.0 ± 0.7 *	3.0 ± 0.6

**Table 3:** Lift-off point ($A_{0}$), collapse pressure ($\pi_{c}$) and maximal ($C_{s,\max}^{-1}$) compression modulus along with the corresponding surface pressure and area per molecule ($\;\pi_{C_{S,\;\max}^{-1}},{\;A}_{C_{s,\;\max}^{-1}}$) for the indicated monolayers.

Monolayer	$A_{0}$ $\left({Å}^{2}\right)$	$\pi_{c}$ (mN/m)	$C_{S,\;max}^{-1}$ (mN/m)	$\pi_{C_{S,\;max}^{-1}}$ (mN/m)	$A_{C_{S,\;max}^{-1}}$ $\left({Å}^{2}\right)$
PL−choline (20 °C)	99	49	108	39	49
PL−choline (37 °C)	108	46	109	38	54
PL+choline (20 °C)	71	48	119	37	39
PL+choline (37 °C)	76	46	116	33	44

## Data Availability

Not applicable.
